# Sex-Dependent Effects of Chronic Social Defeat on Emotional and Social Behaviors, and Parameters of Oxytocin and Vasopressin Systems in Mandarin Voles (*Microtus mandarinus*)

**DOI:** 10.3389/fnins.2021.625116

**Published:** 2021-05-11

**Authors:** Wenjuan Hou, Huan Ma, Yufeng Xun, Xin Zhang, Wenqi Cai, Shuying Huang, Zhixiong He, Fadao Tai, Rui Jia

**Affiliations:** Laboratory for Brain and Behavioral Science, Shaanxi Normal University, Xi’an, China

**Keywords:** sex difference, oxytocin, vasopressin, emotional behavior, social behavior

## Abstract

In the regulation of emotional and social behaviors, both oxytocin (OT) and vasopressin (AVP) are sex specific. Although significant sex differences have been reported in the context of behavioral and hormonal responses to social stress, such differences in response to chronic social defeat stress (CSDS) and the underlying neural mechanisms remain largely unknown. By investigating monogamous mandarin voles (*Microtus mandarinus*), CSDS was found to decrease the percentages of time spent in the central area of the open field, in the open arms of the elevated plus maze, as well as in the light area of the light and dark boxes in both male and female voles. CSDS also increased the observed level of social withdrawal in both sex groups. However, CSDS exposure increased the percentages of immobile time in both the tail suspension test and the forced swim test and reduced the locomotor activity in the open field (in females only). Along with these behavioral changes, the oxytocin receptor (OTR) levels in the nucleus accumbens (NAc) were significantly lower in CSDS-exposed voles of both sexes; however, in males, the levels of OTR in the paraventricular nucleus (PVN) were reduced. CSDS-exposed males showed lower levels of V1aR in the NAc than CSDS-exposed females. Furthermore, induced by a single social defeat event, CSDS reduced c-Fos and OT double labeling in the PVN of females but increased c-Fos and AVP double-labeled neurons in the PVN of males exposed to a single social defeat event. Collectively, the present study indicates that OT and AVP systems may play important regulatory roles in the sex differences of behavioral performances in response to CSDS. These findings suggest mandarin voles as a useful animal model for studying sex-specific behavioral performance and the underlying neurobiological mechanisms of stress-related mental disorders in preclinical studies.

## Introduction

Grievous and stressful life experiences have been reported to increase the risk for depression or anxiety (Klinsey et al., 2007; Franklin et al., 2018). As an etiologically valid stressor, chronic social defeat stress (CSDS) provides a relevant model for investigating the etiology of stress-related disorders (Koolhaas et al., 1999; Steinman and Trainor, 2017). Specifically, the CSDS paradigm has been widely applied as an animal model of depression and anxiety disorders (Blanchard et al., 2001). However, the mechanisms with which these stressful experiences are translated into behaviors are poorly understood, and therefore, the influencing factors should be investigated further. While males and females generally tend to exhibit very similar behaviors, they often use different mechanisms when responding to social and emotional challenges and opportunities (Bangasser and Wicks, 2017; Palanza and Parmigiani, 2017). To understand the mechanisms with which the brain regulates these behaviors, many variables need to be considered, and sex is one of the prominent variables.

Studies have found that different sexes may display different responses when facing social defeat (Bangasser and Wicks, 2017; Steinman and Trainor, 2017). In humans, compared with men, women are more vulnerable to bullying and verbal abuse and are more likely to exhibit depressive, anxious, submissive, and withdrawal behaviors (Kessler et al., 1993, 1995). In rodents, social defeat yields different effects in different sexes, species, and social organizations (Holly et al., 2012; Page et al., 2016; Steinman and Trainor, 2017). For example, male Syrian hamsters (*Mesocricetus auratus*) are more vulnerable to conditioned defeat than females. While male Syrian hamsters exhibit a prolonged behavioral response to conditioned defeat, females remain aggressive or only exhibit a transient submissive response (Huhman et al., 2003). Social defeat has also been identified as a major stressor in male but not in female rats (Haller et al., 1999). In monogamous female California mice (*Peromyscus californicus*) (Trainor et al., 2011; Greenberg et al., 2015) and mandarin voles (*Microtus mandarinus*) (Wang et al., 2018; Hou et al., 2020), social defeat increases anxiety-like responses, induces social withdrawal, and reduces social interaction behaviors. However, the impact of CSDS on both the emotional and social behaviors in monogamous rodents and its underlying mechanisms have not been established to date.

In commonly used laboratory rodents, males show high levels of aggression when competing for resources, mating, and defending their territory (Haller et al., 1999), while females exhibit low levels of interfemale aggression (Holly et al., 2012). In contrast, in a number of rodent species, such as California mice (Silva et al., 2010; Greenberg et al., 2015), mandarin voles (Wang et al., 2018; Hou et al., 2020), and Syrian hamsters (Solomon et al., 2007), females also exhibit interfemale aggression. Since in mandarin voles, both males and females aggressively defend joint territories against intruders, this monogamous species is an ideal experimental animal for studying the sex-specific effects of CSDS (Wang et al., 2018).

One potential mechanism underlying the CSDS-induced responses of emotional and social behaviors may be sex-specific changes in the oxytocin (OT) and arginine vasopressin (AVP) systems. OT and AVP are evolutionarily conserved and are involved in the regulation of mood and diverse social behaviors in a sex-dependent manner (Dumais and Veenema, 2016; Li et al., 2016). Studies about the long-term effect of social defeat indicated that social defeat downregulates the activity of AVP neurons in the caudal paraventricular nucleus (PVN) in male California mice but not in females (Steinman et al., 2015). However, this long-term effect of social defeat on the activity of OT cells in the PVN mainly affected the rostral side, and chronic social defeat decreased the activity of OT-positive cells in female California mice but not in males (Steinman et al., 2016). Moreover, intranasal OT produces sex-specific behavioral effects. For example, intranasal OT largely counteracted the effects of social defeat stress on social interaction behavior in male California mice but had no obvious effect in females (Steinman et al., 2016). AVP elevates anxiety levels in male rats but had no effect on female rats (Landgraf et al., 1995, 2003; Veenema et al., 2010). Activation of the oxytocin receptor (OTR) in the medial prefrontal cortex reduces anxiety levels in male mice and increases social motivation in females (Nakajima et al., 2016). In the rodent brain, both OTR and the vasopressin 1a receptor (V1aR) show distinct, largely non-overlapping, and sex-specific expression patterns. This indicates their sex-specific roles in the modulation of emotional and social behaviors (Smeltzer et al., 2006; Dumais and Veenema, 2016). Specifically, sex differences in the behavioral regulation by OT and AVP suggest that perturbations of these systems may have different roles in males and females (Carter, 2007; De Vries, 2008). Various types of social stresses may exert different effects on the OT and AVP systems in different sexes and ultimately induce distinct behavioral manifestations in the different sexes (Bredewold and Veenema, 2018). Maternal separation is known to decrease the number of OT-immunoreactive (ir) neurons in the PVN in females but not in males (Veenema et al., 2007). Litvin et al. found that CSDS increases the number of activated AVP-ir cells (Litvin et al., 2011). Therefore, the present study predicted that CSDS may alter OT and AVP systems in a sex-dependent manner that results in different alterations of emotional and social behaviors.

Both OT and AVP are key regulators of emotional and social behaviors in specific brain regions (Landgraf and Neumann, 2004; Neumann, 2008; Insel, 2010). OT is predominantly synthesized in the PVN and supraoptic nucleus (SON) and modulates the activation of social behavioral networks such as the lateral septum, bed nucleus of stria terminalis (BNST), and medial amygdala (MeA) (Newman, 1999; Goodson, 2005; Dumais et al., 2013). AVP is predominantly produced in the PVN, SON, suprachiasmatic nucleus, BNST, and MeA, which have been implicated in social behaviors and responses to stress (De Vries and Panzica, 2006; Rood et al., 2013). The nucleus accumbens (NAc) is a key region of the reward circuit and expresses high levels of OTR. Variations in OTR densities in the NAc have been shown to contribute to differences in social behaviors (Ross et al., 2009). Previously, it has been shown that pharmacologically induced OTR activation in the NAc reverses the effects of CSDS on emotional and social behaviors in female mandarin voles (Wang et al., 2018; Hou et al., 2020). Evidence suggests that OT and AVP signaling pathways in the PVN and NAc play critical roles in regulating emotional and social behaviors in different species. However, whether OTR and V1aR levels in the brain regions are responsible for inducing behavioral alterations following CSDS remains to be clarified. Furthermore, whether CSDS induces sex-specific OT and AVP systemic changes in these regions needs to be further elucidated.

By utilizing monogamous mandarin voles, this study investigated sex-specific effects of CSDS on (i) emotional and social behaviors and (ii) the parameters of oxytocinergic and vasopressinergic systems. Experiment 1 assessed sex-specific differences in the alterations of emotional and social behaviors in response to CSDS. In addition, the effects of CSDS on the expression levels of OTR, AVP, and V1aR protein in the NAc and PVN were compared between different sexes. Experiment 2 examined OT, AVP, c-Fos, OT/c-Fos, and AVP/c-Fos immunoreactivity in the PVN (a major region where a large number of neurons of OT and AVP are distributed) after both defeated and undefeated voles were exposed to an unfamiliar aggressive vole of the same sex. Therefore, this study provides important insights into the mechanisms underlying sex differences in the vulnerability and resilience to chronic social stress. These results may help to better understand CSDS-induced sex-specific alterations of social and emotional behaviors.

## Materials and Methods

### Animals

The mandarin voles were obtained from our laboratory colony and were housed in standard polypropylene cages (32.1 × 21.5 × 16.5 cm) containing Aspen shavings and cotton wool as nesting material. Food and water were provided *ad libitum*. The laboratory-reared F3 generation of a wild stock caught in Henan province, China, was used for the experiments. Voles were maintained in a 12/12-h light/dark cycle at temperatures of 23 ± 2°C. Offspring were weaned from breeding pairs at 21 days of age and were housed with more than two same-sex, age-matched littermates and/or unrelated juveniles. A total of 64 naive mandarin voles (80 days of age, 28–35 g in weight) were used for this experiment. All procedures were approved by the Animal Care and Use Committee of Shaanxi Normal University and were conducted in accordance with the Guide for the Care and Use of Laboratory Animals of China.

### Chronic Social Defeat Stress Paradigm

Animals were randomly distributed into a control group (male, *n* = 16; female, *n* = 16) and a CSDS group (male, *n* = 16; female, *n* = 16). To avoid the litter effect within groups, each litter contributed only one offspring per group. The CSDS paradigm was established as described previously (Hou et al., 2020). Briefly, intruder mandarin voles were introduced into the home cage of an unfamiliar aggressor vole and were allowed to interact for 10 min. Voles (with ages ranging from 80 to 120 days) with attack latencies shorter than 30 s on three consecutive daily screening sessions were chosen as aggressors. During the exposure period, the experimental voles (i.e., the intruders) were physically defeated over six attacks by same-sex aggressive voles (i.e., the residents). When they showed submissive postures at the same time, they were separated with a perforated Plexiglas divider to enable sensory contact for the remainder time of the 10 min. If the resident vole did not attack the intruder, the intruder was introduced into the cage of a new aggressor vole. All sessions were monitored to ensure that intruder voles were consistently defeated but were not injured. After 10 min of interaction, the defeated vole was removed from the cage of the resident and was housed alone until the next defeat. After 24 h, the experimental vole was randomly exposed to a new resident vole to prevent habituation. Control voles were housed individually in identical cages but were never exposed to aggressors. The social defeat procedure was continued for 14 consecutive days. Although all sessions were monitored seriously, five voles (four females and one male) were excluded because of severely inevitable injury. The injury ratio was kept as low as possible.

**FIGURE 1:**
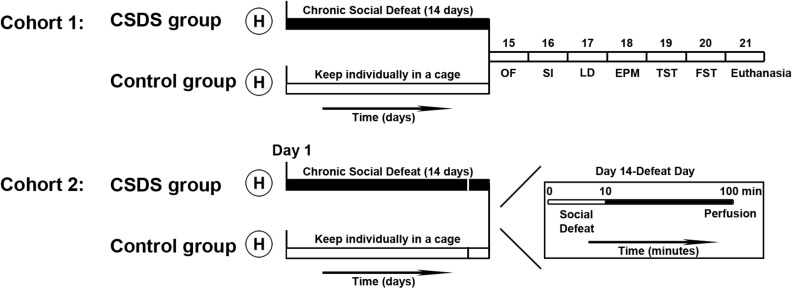
Time course of the experimental procedures performed in this study. After handling (H), all voles were divided into two cohorts. In cohort 1, voles (control group: male, *n* = 10; female, *n* = 10; CSDS group: male, *n* = 10; female, *n* = 10) were individually housed in their own cages or exposed to social defeat for 14 days, after which they were subjected to behavioral tests. Voles (control group: male, *n* = 6; female, *n* = 6; CSDS group: male, *n* = 6; female, *n* = 6) were euthanized after all behavioral tests were completed and brain tissue samples were collected to examine protein levels. In cohort 2, voles (control group: male, *n* = 6; female, *n* = 6; CSDS group: male, *n* = 6; female, *n* = 6) were perfused after 90 min immediately on the 14th episode of social defeat (for the CSDS group) or the first episode of social defeat (for the control group) to process the brains for the immunofluorescence assays (OF, the open field test; SI, the social interaction test; LD, the light and dark test; EPM, the elevated plus maze test; TST, the tail suspension test; FST, the forced swim test).

The design of the experimental procedure is illustrated in Figure 1. All voles were randomly divided into two cohorts. One cohort of voles was used for the behavioral tests (control group: male, *n* = 10, female, *n* = 10; CSDS group: male, *n* = 10, female, *n* = 10). After behavioral tests, some of the animals were used to measure protein expression levels (control group: male, *n* = 6, female, *n* = 6; CSDS group: male, *n* = 6, female, *n* = 6). The other cohort of voles (control group: male, *n* = 6, female, *n* = 6; CSDS group: male, *n* = 6, female, *n* = 6) were sacrificed with an overdose of phenobarbital sodium and were perfused after the last social defeat in case of the CSDS groups and after exposure to one social defeat in case of the control group. Exposure to one social defeat in the control group was applied with the aim of identifying differences in neuronal activity to the same social stimulation between the control and CSDS groups. After perfusion, brains were harvested for immunofluorescence assays.

### Experiment 1: Effects of CSDS on Emotional Behaviors and Sociality

All behavioral experiments were performed between 8:00 and 10:00 AM. To minimize the effect of previous behavioral testing on the result of the next test, the sequence of behavioral tests was designed from least to most stressful. Only one behavioral test was performed per day.

#### Open Field Test

One day after the last episode of social defeat, animals were placed in an open field apparatus (length × width × height, 50 cm × 50 cm × 40 cm, 200 lx) for 5 min in a quiet experimental environment equipped with a digital video camera. Scoring was carried out with the VideoMot2 system (TSE, Bad Homburg, Germany). The percentage of time spent in the central area (i.e., the time spent in the central area/the total test time × 100%) was recorded. The total distance moved in the open field, and the number of entries to the central area were used as indicators of the locomotive levels of voles. At the end of each experiment, 75% alcohol was used to remove residues and odors from the box to prevent these from affecting the next vole.

#### Social Interaction Test

Social interaction testing was performed following a previously described protocol (Wang et al., 2018). The social interaction apparatus consists of a large Plexiglas box (length × width × height, 50 cm × 50 cm × 40 cm, 200 lx) containing a small wire-mesh cage (length × width × height, 10 cm × 10 cm × 15 cm), which was placed against one wall of the apparatus. During the acclimatization phase, each vole (defeated or control) was allowed to move freely and explore the empty cage for 10 min. Then, the vole was placed in the box for 10 min during the second phase, and an age-matched unfamiliar vole was placed into the wire-mesh cage. The video tracking system recorded the time spent inside the interaction zone, which was defined as an area of 600 cm^2^ (length × width, 20 cm × 30 cm) around the cage. The social interaction ratio was calculated, defined as 100 × (interaction time, target present)/(interaction time, target absent). Between sessions, both the cage and the wire box were thoroughly cleaned with 75% alcohol.

#### Light and Dark Test

The apparatus used for the light and dark test was a closed methacrylate box that was separated into two compartments by a methacrylate partition: an enclosed dark compartment (length × width × height, 27 cm × 18 cm × 27 cm, without illumination) and a hyaline bright compartment (length × width × height, 27 cm × 27 cm × 27 cm, with a light intensity of approximately 350 lx). A centrally positioned opening (width × height, 6 cm × 7 cm) within the partition allowed the voles to move back and forth. At the beginning of the test, voles were placed individually at the center of the light compartment, facing away from the door. The percentage of time spent in the light compartment (i.e., the time spent in the bright box/total test time × 100%) was measured for 5 min. At the end of each test, voles were returned to their home cages, and the apparatus was thoroughly cleaned by 75% alcohol.

#### Elevated Plus Maze Test

The elevated plus maze consisted of two open arms (length × width × height, 30 cm × 7 cm × 0.5 cm) and two closed arms (length × width × height, 30 cm × 7 cm × 10 cm) crossing at the midpoint (the central zone, length × width, 7 cm × 7 cm). The experiment was carried out in a controlled light room (200 lx). Facing the closed arm, voles were placed in the central region and were recorded for 5 min with the digital video tracking system. The percentages of time spent in the closed arms (i.e., the time spent in closed arms/the total test time × 100%) and in the open arms (i.e., the time spent in open arms/the total test time × 100%) were recorded. The total distance traveled was measured to assess the movement ability of a subject. The apparatus was cleaned with a 75% alcohol solution between trials.

#### Tail Suspension Test

The tail suspension apparatus consisted of a tail suspension frame and a tail suspension monitor (TSE Systems, Germany). In a quiet environment, a vole was fixed on the suspension frame (using adhesive tape) 1 cm from the tip of the tail for 6 min. The percentage of time spent in an immobile state (i.e., immobility during the last 4 min/total test time × 100%) was recorded and analyzed.

#### Forced Swim Test

Voles were placed in a clean glass cylinder (diameter, 13 cm; height, 24 cm) filled with water (14 cm depth) at a temperature of 25 ± 1°C for 6 min. The degree of immobility-induced behavior was assessed as the time spent floating without struggling while only making necessary movements to keep its head above the water. The immobility time was recorded by a video camera and analyzed using J Watcher software^1^ during the final 4 min of the test. The percentage of time spent in an immobile state (i.e., immobility during the last 4 min/total test time × 100%) was recorded. Water was changed after every trial to remove residues and odors.

### Experiment 2: Effects of CSDS on OT, AVP, OTR, and V1aR Levels

#### Western Blot

The voles (CSDS group: male, *n* = 6; female, *n* = 6; control group: male, *n* = 6; female, *n* = 6) were anesthetized using an overdose of pentobarbital sodium (60 mg/kg) and were sacrificed within 24 h after the end of all behavioral tests (Figure 1). The brains were quickly harvested. Coronal brain sections (300 μm) were cut on a cryostat (VT-1200S, Leica, Wetzlar, Germany). Samples from the NAc and PVN were bilaterally obtained using a 1-mm diameter puncher under a dissecting microscope in reference to brain atlases (The Mouse Brain in Stereotaxic Coordinates, second edition, by George Paxinos and Keith B.J. Franklin). Samples were frozen at −80°C. The NAc and PVN of voles were defined as anteroposterior (AP): 1.78–0.86 mm and −0.58 to −1.22 mm, respectively. Radioimmunoprecipitation assay (RIPA) buffer (10,000 μl/g) and protease inhibitors (100 μl/g) were added, and cells were crushed by an ultrasonic cell crusher (JY96-IIN, Scientz Biotechnology, Ningbo, China). After centrifugation at 12,000 rpm for 15 min at 4°C, the supernatant was removed, and the total protein concentration was measured using the bicinchoninic acid (BCA) protein assay kit (PA115, Tiangen Biotech Co., Ltd., Beijing, China). The concentration of each sample was diluted to 2 μg/ul by adding 5 × loading buffer and RIPA lysis buffer.

Then, 10 μl of the sample was loaded in each well. Samples were separated using 10% sodium dodecyl sulfate–polyacrylamide gel electrophoresis (SDS-PAGE) gels and were transferred to a polyvinylidene fluoride (PVDF) membrane at 4°C, at 100 V constant voltage for 90 min. Membranes were blocked with 5% of dried skimmed milk at 25°C for 90 min and were incubated with rabbit anti-OTR, rabbit anti-AVP, goat anti-V1aR, or mouse anti-β-tubulin primary antibodies (Table 1) overnight at 4°C. These antibodies were used appropriately and were previously validated in mandarin voles (He et al., 2019; Hou et al., 2020; L. F. Li et al., 2019; Yuan et al., 2019, 2020). Following overnight incubation, the membranes were rinsed and incubated with goat antirabbit, goat antimouse, or rabbit antigoat secondary antibodies (Table 1) at 25°C for 1 h. A fully automatic chemiluminescence image analysis system (Tanon 6200 Luminescent Imaging Workstation, Tanon, Shanghai, China) was used to visualize and photograph the protein bands. The protein bands were quantitatively analyzed using ImageJ software (NIH, Bethesda, MD, United States), and the house-keeping gene encoding β-tubulin was used as the internal control.

**TABLE 1:** Antibodies used in Western blotting and immunofluorescence.

Target	Host species/conjugate	Vendor	Dilution	
***Primary antibodies***				

Oxytocin receptor	Rabbit (IgG)	Abcam, UK (Cat#: ab181077)	WB:	1:2000
Arginine vasopressin	Rabbit (IgG)	Millipore, Germany (Cat#: AB1565)	WB:	1:4000
			Immuno:	1:4000
Arginine vasopressin 1a receptor	Goat (IgG)	Gene Tex, USA (Cat#: GTX89114)	WB:	1:4000
β-Tubulin	Mouse (IgG)	CWBIO, CNN (Cat#: CW0098)	WB:	1:4000
Oxytocin	Mouse (IgG)	Millipore, Germany (Cat#: MAB5296)	Immuno:	1:7500
c-Fos	Rabbit (IgG)	Abcam, UK (Cat#: Ab190289)	Immuno:	1:2000

***Secondary antibodies***				

Mouse IgG	Peroxidase-conjugated goat antimouse	Santa Cruz Biotechnology, USA (Cat#: sc-358914)	WB:	1:10,000
	Antimouse goat antibody conjugated with Dylight 488	BOSER, CNN (Cat#: BA1126)	Immuno:	1:200
Rabbit IgG	Peroxidase-conjugated goat antirabbit	ZSGB-BIO, CNN (Cat#: ZB-2301)	WB:	1:10,000
	Antirabbit goat conjugated with TRITC	Jackson ImmunoResearch, USA (Cat#: ZB-2301)	Immuno:	1:400
Goat IgG	Peroxidase-conjugated rabbit antigoat	ZSGB-BIO, CNN (Cat#: ZB-2306)	WB:	1:10,000

#### Double Immunofluorescence for c-Fos With OT or AVP

To explore changes in the activity of neurons in specific brain regions of mandarin voles exposed to CSDS (CSDS group: male, *n* = 6; female, *n* = 6) or after the initial episode of social defeat (control group: male, *n* = 6; female, *n* = 6), voles were anesthetized with an overdose of pentobarbital sodium (60 mg/kg) after 90 min (Figure 1). Then, voles were transcardially perfused with 0.01 M of phosphate-buffered saline (PBS) at a pH of 7.4, followed by 4% of paraformaldehyde. After perfusion, the brain was dissected from the skull and postfixed in paraformaldehyde at 4°C for 1 week. Then, the brain was dehydrated in 20 and 30% sucrose solution in PBS until the brain sank to the bottom. The brain was sliced into coronal pieces of 40 μm thickness using a freeze microtome (CM-1850, Leica, Wetzlar, Germany). Every two sections from the serial sections were processed for double immunofluorescence of c-Fos with OT or AVP, as previously described (Veenema et al., 2007; Steinman et al., 2016; He et al., 2019). In brief, coronal sections were incubated in 0.3% hydrogen peroxide (H_2_O_2_) and permeabilized with 0.1% Triton X-100/PBS for 30 min. Sections were then blocked with 5% normal goat serum (AR0009, Boster Bioengineering Co., Ltd., Wuhan, China) for 30 min. For double immunofluorescence staining against c-Fos with OT, sections were incubated overnight with polyclonal rabbit anti-c-Fos antibody (Table 1) and monoclonal mouse anti-OT antibody (Table 1) at 4°C. This was followed by Rhodamine (TRITC) AffiniPure goat antirabbit IgG antibody (Table 1) and Dylight 488-conjugated goat antimouse IgG antibody (Table 1) for 1 h at room temperature. For the double-labeling of c-Fos with AVP, sections were incubated overnight with polyclonal rabbit anti-c-Fos antibody at 4°C. The next day, sections were incubated with TRITC AffiniPure goat antirabbit IgG antibody for 1 h at room temperature in the dark. After rinsing three times in PBS for 5 min each, sections were incubated overnight with polyclonal rabbit anti-AVP antibody (Table 1) at 4°C. On the third day, sections were incubated with Dylight 488-conjugated goat antirabbit IgG antibody (Table 1) for 1 h at room temperature in the dark. 4′,6-diamidino-2-phenylindole (DAPI) (AR1177, Boster Bioengineering Co., Ltd., Wuhan, China) was applied for 10 min for nuclear counterstaining.

#### Quantitative Analysis of c-Fos, OT, and AVP Positive Neurons

C-Fos-ir, OT-ir, and AVP-ir neurons were observed, and images were obtained using a fluorescence microscope (Nikon Eclipse 80i, Nikon Instruments, Tokyo, Japan). In reference to the mice brain atlas, the PVN (200-fold magnification) was identified, and positive neurons were counted bilaterally. ImageJ software was used to generate boxes for the PVN (0.35 × 0.43 mm) (Duque-Wilckens et al., 2018; Hou et al., 2020). Every fourth section of the tissue that encompasses the anterior to posterior extent of the PVN (bregma −0.58 to −1.22) was used, and the number of c-Fos-ir, OT-ir, and AVP-ir cells was counted. Positive cells were counted for every single channel (green or red). For analyses of neuronal activation, a single PVN section with the highest number of c-Fos-ir neurons [corresponding to the presence of OT-ir or AVP-ir neurons; merged channels (yellow)] was chosen for quantification. The percentage of colocalization of c-Fos with OT or AVP neurons (i.e., the number of c-Fos with OT or AVP colocalization cells/total OT or AVP cells × 100%) was analyzed as a percentage of activated OT or AVP neurons. The quantification was performed by an observer who was blinded to the experimental conditions.

### Statistical Analyses

All data were analyzed using SPSS 20.0 statistical software (SPSS Inc., Chicago, United States). Two-way analysis of variance (ANOVA) was used to analyze the effects of treatment (control vs. CSDS) and sex (males and females). Simple effects analyses with Bonferroni correction were used when significant interactions were found. Planned comparison t-tests were used as appropriate when the main effects were significant without significant interactions (Anacker et al., 2018; Fang et al., 2018; Walsh et al., 2018). The normality of data and homogeneity of variance were assessed via Q–Q plots and Levene’s test. Data are presented as mean ± SEM, and the significance level was set to *p* < 0.05.

## Results

### Effects of CSDS on Behaviors in the OF, EPM, and LD in Male and Female Mandarin Voles

**FIGURE 2:**
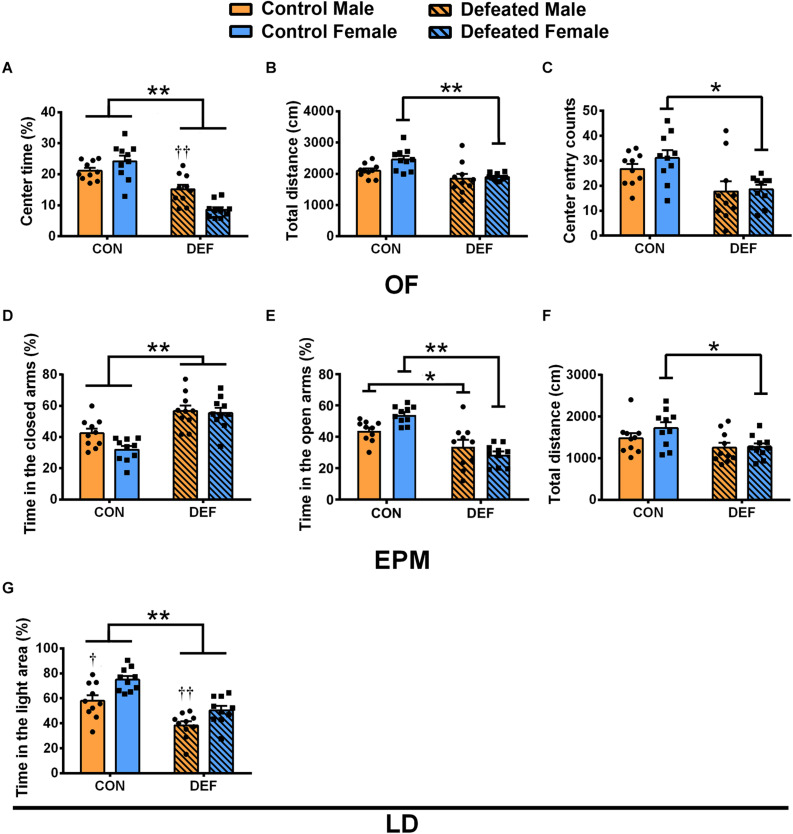
Effects of chronic social defeat stress (CSDS) on behaviors in the open field test (OF), elevated plus maze test (EPM), and light and dark test in male (*n* = 10 control voles, *n* = 10 CSDS voles) and female (*n* = 10 control voles, *n* = 10 CSDS voles) mandarin voles. The percentage of **(A)** time spent in the central area, **(B)** total distance moved, and **(C)** center entry counts in the OF. The percentage of time spent in the **(D)** closed arms and **(E)** open arms and **(F)** total distance moved in the EPM. **(G)** The percentage of time spent on the light side of the LD. Graphs display mean ± SEM. *p < 0.05, **p < 0.01, effect of stress; ^†^*p* < 0.05, ^††^*p* < 0.01, effect of sex; two-way ANOVA (factors sex × treatment).

In the OF, a significant interaction between sex and treatment was found as indicated by the percentage of time spent in the central area [*F*(1,36) = 13.07, *p* < 0.01] (Figure 2A). CSDS caused a significant decrease in the percentage of time spent in the central area compared with the controls in both males (*p* < 0.01) and females (*p* < 0.01); furthermore, sex differences were observed between males and females in CSDS groups (CSDS male versus female: *p* < 0.01) (Figure 2A). In addition, only females that were exposed to CSDS showed a statistically significant decrease in the total distance [main effect of treatment: *F*(1,36) = 15.23, *p* < 0.01; planned comparison t-test, female control versus CSDS: *p* < 0.01] (Figure 2B) and the number of entries into the central area [main effect of treatment: *F*(1,36) = 13.88, *p* < 0.01; planned comparison t-test, female control versus CSDS: *p* < 0.01] (Figure 2C) compared with the control group. These data further demonstrated that there were no sex differences and sex × treatment interaction in the total distance [*F*(1,36) = 3.788, *p* = 0.059; *F*(1,36) = 2.146, *p* = 0.152, respectively] (Figure 2B) as well as in the number of entries to the central area [*F*(1,36) = 0.843, *p* = 0.365; *F*(1,36) = 0.368, *p* = 0.548, respectively] (Figure 2C).

The findings of the EPM test showed that CSDS caused a significant increase in the percentage of time spent in the closed arms in both male and female voles [main effect of treatment: *F*(1,36) = 38.62, *p* < 0.01] (Figure 2D). Two-way ANOVA demonstrated no main effect of sex [*F*(1,36) = 3.783, *p* = 0.06] and an insignificant interaction between sex and treatment [*F*(1,36) = 2.298, *p* = 0.138] (Figure 2D). Moreover, a significant interaction of sex and treatment was found on the percentage of time spent in the open arms [*F*(1,36) = 7.46, *p* < 0.01] (Figure 2E). CSDS decreased the percentage of time spent in the open arms compared with the controls in both males (*p* < 0.05) and females (*p* < 0.01) (Figure 2E). The data showed a decrease in total distances traveled in female voles only [main effect of treatment: *F*(1,36) = 5.922, *p* < 0.05; planned comparison t-test, female control versus CSDS: *p* < 0.05] (Figure 2F). There was neither a main effect for sex [*F*(1,36) = 2.557, *p* = 0.119] nor an interaction effect of sex and treatment [*F*(1,36) = 0.251, *p* = 0.619] for the total distances.

In the LD test (Figure 2G), CSDS decreased the percentage of the time spent in the light area in both males and females [main effect of treatment: *F*(1,36) = 38.29, *p* < 0.01]. Moreover, compared with females, the percentage of time spent in the light area was lower for males in both the control and CSDS groups [main effect of sex: *F*(1,36) = 16.24, *p* < 0.01; planned comparison t-test, control males versus females: *p* < 0.05; defeated males versus female: *p* < 0.01]. There was no sex × treatment interaction difference in the percentage of time spent in the light area [*F*(1,36) = 0.462, *p* = 0.501].

### Effects of CSDS on Behaviors in the TST and FST in Male and Female Mandarin Voles

**FIGURE 3:**
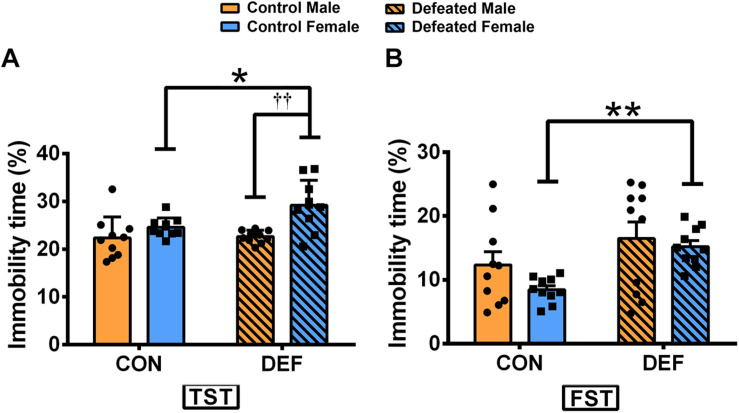
Effects of chronic social defeat stress (CSDS) on behaviors in the tail suspension test (TST) and forced swim test (FST) in male and female mandarin voles. CSDS increased the percentage of immobility time in the **(A)** TST and the **(B)** FST in female mandarin voles (*n* = 10 control voles, *n* = 10 CSDS voles) but did not affect stressed male mandarin voles (*n* = 10 control voles, *n* = 10 CSDS voles). Graphs display mean ± SEM. *p < 0.05, **p < 0.01, effect of stress; ^††^*p* < 0.01, effect of sex; two-way ANOVA (factors sex × treatment).

In the TST (Figure 3A), CSDS only increased the percentage of immobile time in females [main effect of treatment: *F*(1,36) = 4.582, *p* < 0.05; planned comparison t-test, female control versus CSDS: *p* < 0.05]. Moreover, CSDS-exposed female voles displayed higher levels of immobility time than male mandarin voles subjected to CSDS [main effect of sex: *F*(1,36) = 14.63, *p* < 0.01; planned comparison t-test, defeated male versus female: *p* < 0.01]. There was no sex × treatment interaction in the percentage of immobility time [*F*(1,36) = 3.643, *p* = 0.064].

In the FST (Figure 3B), CSDS significantly increased the percentage of immobility in females but not in males [main effect of treatment: *F*(1,36) = 6.572, *p* < 0.05; planned comparison t-test, female control versus CSDS: *p* < 0.01]. There were no main effects for sex [*F*(1,36) = 1.292, *p* = 0.263] and also for sex × treatment interaction effect [*F*(1,36) = 0.126, *p* = 0.725] in the percentage of immobility time.

### Effects of CSDS on the Level of Social Interaction Ratios in Male and Female Mandarin Voles

**FIGURE 4:**
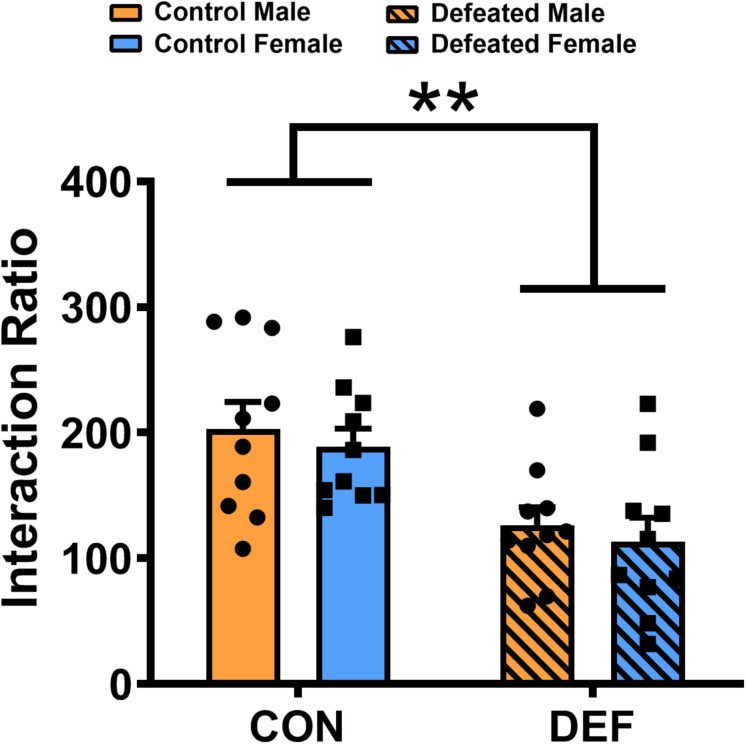
Chronic social defeat stress (CSDS) exposure decreased the social interaction ratio in both male (*n* = 10 control voles, *n* = 10 CSDS voles) and female (*n* = 10 control voles, *n* = 10 CSDS voles) mandarin voles. Graphs display mean ± SEM. **p < 0.01, effect of treatment; two-way ANOVA (factors sex × treatment).

To determine whether the social interactions of male and female voles differ after exposure to CSDS, both sexes were exposed either to an empty cage or a novel vole of the same sex and age (Figure 4). Two-way ANOVA identified a significant impact of CSDS on the social interaction ratio [main effect of treatment: *F*(1,36) = 18.558, *p* < 0.01] in mandarin voles. Voles exposed to CSDS had a significantly lower social interaction ratio compared with both male and female control mandarin voles. There was no main effect for sex [*F*(1,36) = 0.596, *p* = 0.445] and no interaction effect of treatment × sex [*F*(1,36) = 0.001, *p* = 0.973] in terms of the social interaction ratio.

### Effects of CSDS on OTR, V1aR, and AVP Protein Levels in Male and Female Mandarin Voles

**FIGURE 5:**
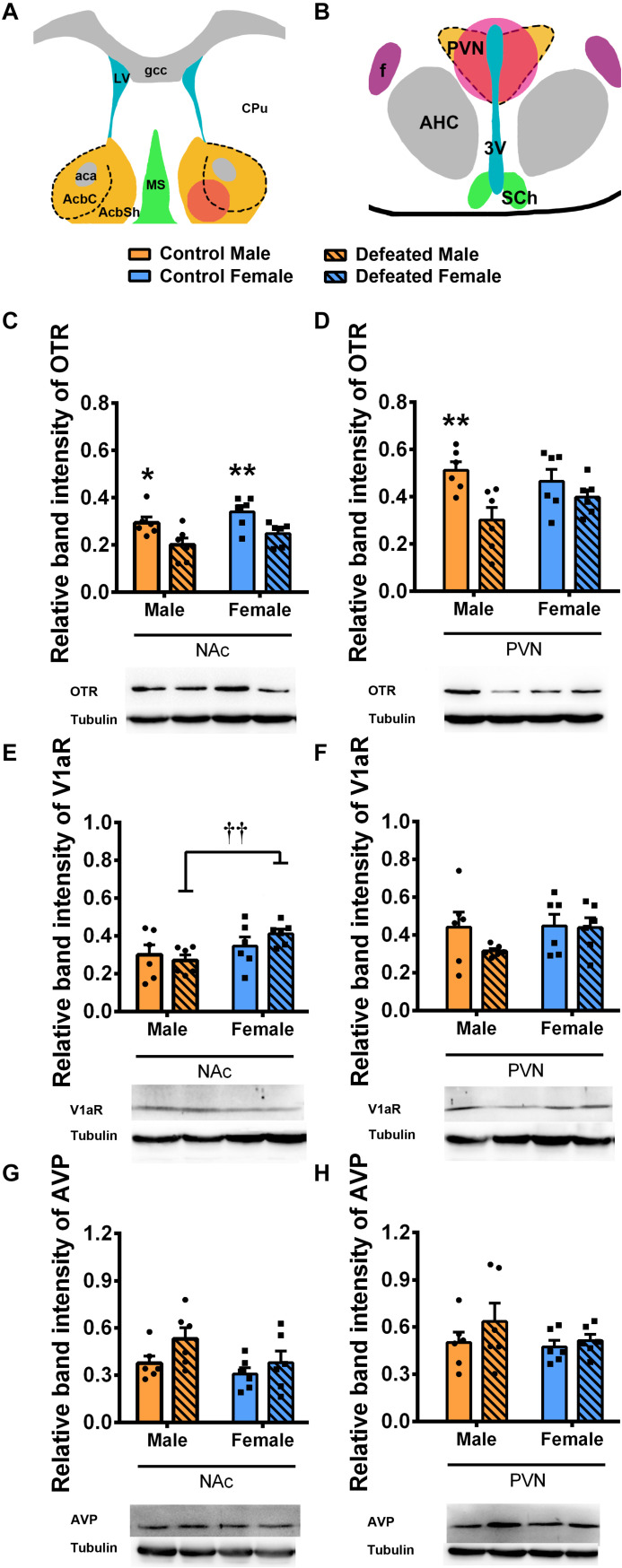
Effect of chronic social defeat stress (CSDS) on **(C,D)** OTR, **(E,F)** V1aR, and **(G,H)** AVP protein levels in the NAc and PVN in both male (*n* = 6 control voles, *n* = 6 CSDS voles) and female (*n* = 6 control voles, *n* = 6 CSDS voles) mandarin voles. Schematic drawings illustrate tissue punch locations in the **(A)** NAc and **(B)** PVN. The red circles show target areas. Graphs display mean ± SEM. *p < 0.05, **p < 0.01, effect of treatment, ††*p* < 0.01, effect of sex; two-way ANOVA (factors sex × treatment). LV, lateral ventricle; aca, anterior commissure; CPu, caudate putamen (striatum); gcc, genu of the corpus callosum; AcbC, the core of nucleus accumbens; AcbSh, the shell of nucleus accumbens; MS, medial septal nucleus; PVN, paraventricular nucleus; f, fornix; AHC, anterior hypothalamic area, central part; 3V, 3rd ventricle; SCh, suprachiasmatic nucleus; NAc, nucleus accumbens; OTR, oxytocin receptor; V1aR, vasopressin 1a receptor; AVP, arginine vasopressin.

Figures 5A,B are schematic drawings which illustrate tissue punch locations in the NAc and PVN respectively. For the OTR, after exposure to CSDS, both male and female voles had lower levels of the OTR in the NAc than the control group [main effect of treatment: *F*(1,20) = 17.722, *p* < 0.01] (Figure 5C). In males, OTR levels in the PVN were significantly decreased after exposure to CSDS but not in females [main effect of treatment: *F*(1,20) = 10.148, *p* < 0.01] (Figure 5D). There were no significant sex × treatment interactions and main effect of sex on the protein level of OTR in the NAc [*F*(1,20) = 0.031, *p* = 0.863; *F*(1,20) = 2.874, *p* = 0.106, respectively] (Figure 5C) and PVN [*F*(1,20) = 2.702, *p* = 0.116; *F*(1,20) = 0.331, *p* = 0.571, respectively] (Figure 5D).

For the V1aR, a significantly lower level of V1aR expression was found in the NAc of males compared with females after exposure to CSDS [main effect of sex: *F*(1,20) = 5.472, *p* < 0.05; planned comparison t-test, defeated male versus female: *p* < 0.01] (Figure 5E). There were no main effects for treatment [*F*(1,20) = 0.192, *p* = 0.666] and no sex × treatment interaction effects [*F*(1,20) = 1.412, *p* = 0.249] for V1aR protein levels in the NAc (Figure 5E). In the PVN, there were no main effects for either sex [*F*(1,20) = 1.31, *p* = 0.266] or treatment [*F*(1,20) = 1.358, *p* = 0.258] and no interaction effect of these factors [*F*(1,20) = 1.032, *p* = 0.322] in terms of V1aR protein levels (Figure 5F).

For the protein level of AVP, no significant effects of sex, treatment, or sex × treatment interaction were found for protein levels in both the NAc [*F*(1,20) = 0.485, *p* = 0.494; *F*(1,20) = 3.679, *p* = 0.069; *F*(1,20) = 3.47, *p* = 0.077, respectively] (Figure 5G) and PVN [*F*(1,20) = 1.036, *p* = 0.321; *F*(1,20) = 1.403, *p* = 0.25; *F*(1,20) = 0.41, *p* = 0.529, respectively] (Figure 5H).

### Effects of CSDS on the Numbers of OT, AVP, OT/c-Fos, and AVP/c-Fos Protein Immunoreactivity Neurons in the PVN

**FIGURE 6:**
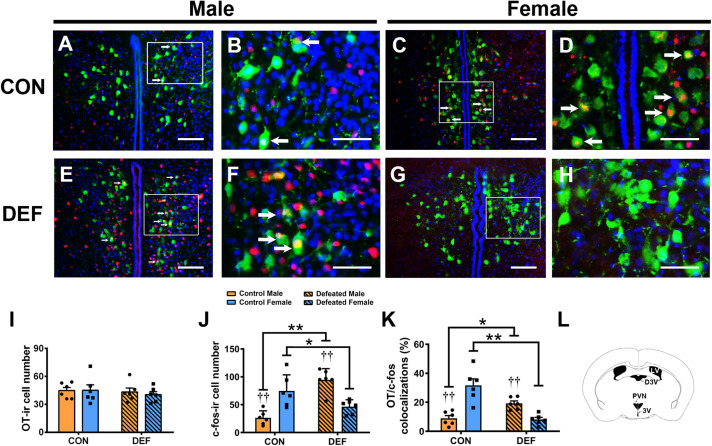
Effects of chronic social defeat stress (CSDS) on OT-ir neurons and OT/c-Fos immunoreactivity neurons in the PVN in both male (*n* = 6 control voles, *n* = 6 CSDS voles) and female (*n* = 6 control voles, *n* = 6 CSDS voles) mandarin voles. **(A–H)** Photomicrographs of immunofluorescence in the PVN. Green = OT; Red = c-Fos; blue = DAPI. White arrows indicate OT/c-Fos double-labeled cells. **(I)** CSDS did not change OT-ir cell numbers in either male or female mandarin voles. **(J)** CSDS impacted on the number of c-Fos-ir cells in both male and female mandarin voles. **(K)** CSDS had an effect on OT/c-Fos colocalization in both males and females. **(L)** Schematic drawing showing the region of the PVN. **(A,C,E,G)** Scale bar = 100 μm. **(B,D,F,H)** Scale bar = 200 μm. LV, lateral ventricle; D3V, dorsal 3rd ventricle; PVN, paraventricular nucleus; 3V, 3rd ventricle. Graphs display mean ± SEM. *p < 0.05, **p < 0.01, effect of treatment. ^††^*p* < 0.01, effect of sex; two-way ANOVA (factors sex × treatment).

**FIGURE 7:**
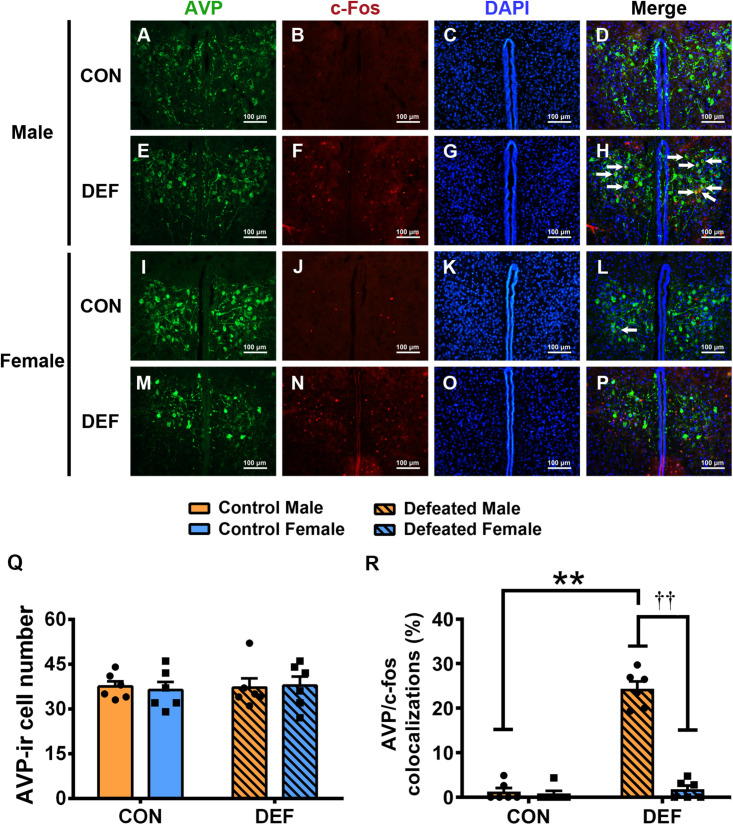
Effects of chronic social defeat stress (CSDS) on numbers of AVP-ir neurons and AVP/c-Fos immunoreactivity neurons in the PVN in both male (*n* = 6 control voles, *n* = 6 CSDS voles) and female (*n* = 6 control voles, *n* = 6 CSDS voles) mandarin voles. **(A–H)** Representative photomicrographs showing AVP and c-Fos cells in the PVN of **(A–D)** control and **(E–H)** CSDS male voles; white arrows in **(H)** indicate AVP/c-Fos double-labeled cells. **(I–P)** Representative photomicrographs demonstrating AVP and c-Fos colocalization in the PVN of **(I–L)** control and **(M–P)** CSDS female voles. Green = AVP; red = c-Fos; blue = DAPI. White arrows indicate AVP/c-Fos double-labeled cells. Scale bar = 100 μm. **(Q)** CSDS did not change AVP-ir cells numbers in either male or female mandarin voles. **(R)** CSDS increased the number of AVP/c-Fos colocalization neurons only in males. Graphs display mean ± SEM. **p < 0.01, effect of treatment. ^††^*p* < 0.01, effect of sex; two-way ANOVA (factors sex × treatment).

To confirm the effects of CSDS on the activities of OT-ir (Figure 6) and AVP-ir neurons (Figure 7), immunofluorescent staining was used to identify protein immunoreactivities of c-Fos, OT, and AVP. Analyses of the numbers of OT-ir neurons showed no main effects for either sex [*F*(1,20) = 0.086, *p* = 0.773] or treatment [*F*(1,20) = 0.521, *p* = 0.479]. Moreover, no interaction effect of these factors [*F*(1,20) = 0.137, *p* = 0.715] were observed in OT-ir cells in the PVN (Figure 6I). In addition, neither treatment [*F*(1,20) = 0.047, *p* = 0.831] nor sex [*F*(1,20) = 0.009, *p* = 0.927] affected the number of AVP-ir cells (Figure 7Q). Furthermore, no interactions between treatment and sex [*F*(1,20) = 0.116, *p* = 0.737] were found in the number of AVP-ir cells.

Two-way ANOVA showed a significant sex × treatment interaction [*F*(1,20) = 35.51, *p* < 0.01] in the number of c-Fos-ir cells (Figure 6J). Interestingly, CSDS-exposed male voles had more c-Fos-ir cells than control males exposed to a single social defeat (*p* < 0.05). However, females exposed to CSDS displayed fewer c-Fos-ir neurons compared with females exposed to a single social defeat (*p* < 0.01). Moreover, in control groups, a lower number of c-Fos-ir cells was observed in males when compared with females after exposure to a single social defeat (*p* < 0.01). Furthermore, males displayed more c-Fos-ir neurons compared with females after exposure to CSDS (*p* < 0.01).

A significant interaction was found between treatment and sex on the percentage of OT/c-Fos-positive cells [*F*(1,20) = 40.03, *p* < 0.01] (Figure 6K). CSDS significantly decreased the percentage of OT/c-Fos-positive neurons in females (*p* < 0.01) but significantly increased it in males (*p* < 0.05). Moreover, CSDS exhibited marked sex differences in the percentage of OT/c-Fos-positive neurons. A single instance of social defeat resulted in a decrease in OT/c-Fos-positive neurons in males compared with females of the control group (*p* < 0.01). The percentage of OT/c-Fos-positive neurons in CSDS-exposed males was significantly higher than that of females (*p* < 0.01).

For the percentage of AVP/c-Fos positive neurons (Figure 7R), a significant treatment × sex interaction was found [*F*(1,20) = 101.897, *p* < 0.01]. Male voles exposed to CSDS had a higher percentage of AVP/c-Fos colocalization than the control group (*p* < 0.01) or females exposed to CSDS (*p* < 0.01). The statistical data indicated that no significant sex differences existed between the two control groups (*p* = 0.736). In addition, no significant differences were found between the control group and the CSDS groups in females (*p* = 0.503).

## Discussion

For these experiments, mandarin voles were used to investigate emotional and social behaviors associations with OT and AVP after CSDS exposure. A number of significant sex-specific differences were found in the effect of CSDS on emotional and social behaviors. Furthermore, the obtained findings suggest sex-specific impacts of OT and AVP systems in the NAc and PVN on emotional and social behavioral changes induced by CSDS.

### Sex Differences in Emotional and Social Behaviors Induced by Chronic Social Defeat Stress

In the present study, CSDS decreased the percentages of time spent in the central area of the OF, the open arms of the EPM, and the light area of the LD. The percentage of time spent in the closed arms of the EPM was increased in both male and female mandarin voles. These findings are consistent with previous investigations in male rodents (Klinsey et al., 2007; Venzala et al., 2012; Hollis and Kabbaj, 2014). However, in this study, only females exhibited an increased percentage of immobility time in the TST and the FST and a decreased total distance in the OF and EPM tests induced by CSDS. These results indicate that females are more susceptible to chronic stress than males (Pierrehumbert et al., 2010; Holt-Lunstad et al., 2011), and CSDS only reduced the levels of locomotor activity in females. In general, males were more aggressive and interactive when encountering another aggressive individual, while females displayed social avoidance and low aggression (Lebron-Milad and Milad, 2012; Cavigelli and Caruso, 2015; Weller et al., 2019). This indicates that both sexes have different coping mechanisms for social defeat (Steinman and Trainor, 2017). Similarly, a previous study suggested that the reduced conspecific social interactions of female rats compared with their male counterparts is possibly the result of higher social anxiety in female rats (Carrier and Kabbaj, 2012). When female rats were exposed to foot shock stress, they generated fear memories associated with novel environments more robustly and for a longer period of time than their male counterparts (Lynch et al., 2013). These results are inconsistent with previous reports that female rats exposed to chronic mild stress exhibited more active behaviors in the FST compared with male rats (Dalla et al., 2005). This may be due to the different susceptibilities to chronic stress between sexes, as well as the different stressors.

The results of the present study showed that male and female mandarin voles subjected to CSDS become more aversive to social stimuli compared with control animals. This is a classic behavioral response induced by chronic stress that affects both sexes (Venzala et al., 2012; Steinman and Trainor, 2017). This response reduces the motivation to engage in social stimuli (Trainor et al., 2011). Similar to the findings of the present study, it has been confirmed that social defeat stress significantly induces social withdrawal and social vigilance, which are common psychiatric phenomena associated with anxiety, depression, and other emotional disorders (Duque-Wilckens et al., 2018). A previous study by Wang et al. also documented that female mandarin voles showed marked social avoidance behavior in response to CSDS exposure (Wang et al., 2018).

### Sex Differences in the OT and AVP Systems in Response to CSDS

To assess sex differences among the effects of CSDS on OT-ir and AVP-ir neurons on PVN activation, c-Fos was stained in OT or AVP cells. An interesting finding is that CSDS decreased the number of OT/c-Fos double-labeled neurons in the PVN of females, but an increase in the number of OT/c-Fos double-labeled neurons in the PVN induced by CSDS was observed in males. Female CSDS-exposed voles had fewer OT/c-Fos double-labeled neurons compared with males exposed to CSDS. Previous studies have documented that social isolation elevates the neural activation of OT cells in both male and female prairie voles (Grippo et al., 2009). This may be because CSDS is a different stress paradigm than chronic social isolation and may exert its effect on the activation level of OT neurons in the PVN via different neurobiological mechanisms. Control females that were subjected to a single defeat event had more OT/c-Fos double-labeled neurons compared with males. In contrast, an increased number of OT/c-Fos cells was only observed in male California mice after a single social defeat event (Steinman et al., 2016). The stress-buffering role of OT has been widely proven (Nomura et al., 2003; Takayanagi et al., 2005). Organisms may increase endogenous OT levels by enhancing the reactivity of the OT system to coordinate or inhibit behavioral or physiological responses to acute stress. Loss of the OT buffering effect, caused by the observed reduction of OT neuronal activation, may also contribute to sensitive behavioral responses to CSDS in females. In addition, the fact that the female HPA axis response is more sensitive to stress than the male HPA axis response (Ehlers et al., 1993; McCormick et al., 2005) may be another reason why females are more vulnerable to chronic stress and are thus more likely to display susceptible behavioral responses.

CSDS increased AVP/c-Fos double-labeled neurons in the PVN of males but did not affect females. Previous findings have shown that male rodents exposed to CSDS exhibited elevations in AVP/c-Fos double-labeled neurons in the PVN (Litvin et al., 2011). CSDS-exposed males had more neurons with AVP/c-Fos colocalizations compared with CSDS-exposed females. A previous study showed that AVP increases anxiety levels in males, as assessed by the EPM test, but did not affect females (Bredewold et al., 2014). Microinjection of the antisense oligodeoxynucleotide of the V1 vasopressin receptor into the septum reduced anxiety-related behavior levels in rats (Landgraf et al., 2003). This anxiolytic effect of AVP in male rats has also been reported by other studies (Liebsch et al., 1996; Everts and Koolhaas, 1999). Thus, these studies support the suggestion that the observed behavioral changes of male mandarin voles may be due to an increased activity of AVP neurons in the PVN, induced by CSDS.

The present study showed that CSDS reduced the OTR levels in the NAc in both male and female mandarin voles. It has been previously shown that female mandarin voles exposed to CSDS exhibit a decreased density of OTR in the NAc and OT-ir fibers in the shell of the NAc (Hou et al., 2020). Several studies have also shown that experiencing stress causes a decrease in OTR in the NAc (Bahi et al., 2016; Bosch et al., 2016; Donovan et al., 2018). For example, immobilization stress decreases OTR expression in the NAc in prairie voles (Donovan et al., 2018). The NAc is a key region of the brain that plays a central role in the pathogenesis of psychiatric disorders (Disner et al., 2011). In monogamous male prairie voles, in the NAc, OT is involved in coping with the stress of losing a partner (Bosch et al., 2016). A decrease in OTR binding in the NAc (induced by immobilization) resulted in increased levels of anxiety-like behavior (Donovan et al., 2018). Moreover, social isolation resulted in lower levels of OTR in the NAc of female rats (Oliveira et al., 2019).

Another interesting finding of the present study is that CSDS decreased the PVN OTR levels only in males but not in females. Males subjected to CSDS had lower levels of V1aR expression in the NAc than females subjected to CSDS. OTR and V1aR are co-expressed in brain regions such as the PVN of the hypothalamus and are remarkably complementary (Raggenbass et al., 1989; Insel and Shapiro, 1992; Stoop, 2012). Structurally, AVP differs from OT at only two amino acid positions (Young, 1999). Both OT and AVP systems interact, thus modulating the responses to chronic stress in males. AVP binds to the OTR in the PVN and consequently induces negative feedback that reduces the release of AVP. It can be inferred that the reduction in OTR levels in the PVN can eliminate the negative feedback and result in high levels of AVP. This in turn causes higher levels of anxiety in males. Previous study has showed that dominant male and female hamsters display similar levels of aggression, but the V1aR density in the anterior hypothalamus of dominant males was higher than in dominant females. Subordinate males display higher levels of submissive behavior and lower levels of V1aR binding than subordinate females. This finding suggests that V1aR levels in the anterior hypothalamus play different roles in mediating both dominance and aggression in different sexes (Grieb et al., 2020). Social isolation has different effects on V1aR and OTR binding in different brain regions and different sexes, which may be associated with increases in aggressive behavior induced by social stress in male and female hamsters (A. P. Ross et al., 2019). These findings suggest that CSDS may alter the levels of V1aR and OTR and may also alter their crosstalk in a sex- and region-dependent way. This subsequently affects emotional and social behaviors in both sexes.

In the future, more detailed studies are needed to better determine sex differences in the OT and AVP systems. In particular, differences in the OT-ir cells in the BNST and SON should be investigated, which are also important for the modulation of behavioral responses to stress (O’Connell and Hofmann, 2012), and OTR binding in brain regions such as the MeA and the lateral septum (Dumais et al., 2013). AVP projections from the PVN, SON, suprachiasmatic nucleus, and MeA have functionally distinct roles. It is therefore crucial to identify the site of origin of AVP-ir fibers (De Vries, 2008; Rood et al., 2013). The present study verified the level of AVP-ir cell activation in the PVN affected by CSDS. AVP innervation functions in other brain regions should be elucidated in future studies. Moreover, the exact and detailed links between cellular alteration in the OT and AVP systems and behavioral outcomes remain unclear. In addition, whether the PVN-NAc oxytocinergic projection is selectively involved in the modulation of CSDS-induced stress responses remains unknown. The cause–effect relationship should be investigated further using additional techniques, such as pharmacological or optogenetic methods. Besides, the presented results only indicated sexual differences in changes in the levels of a particular social approach. Further studies are required to assess complex social behaviors such as social vigilance. It should also be pointed out that the CSDS paradigm that was used in this study made it difficult to discriminate between the different effects of physiological and psychological stress. Therefore, improved CSDS models need to be developed in the future to overcome this limitation.

## Conclusion

In conclusion, both male and female mandarin voles displayed changes in emotional behaviors as well as increased social avoidance when exposed to CSDS. However, only CSDS-exposed females showed increased negative coping behaviors and decreased locomotor activity. Changes in the OTR in both sexes suggests the involvement of the OTR in the NAc in the mediation of emotional alterations and decreased sociality as induced by CSDS. Elevated activation of AVP cells in the PVN in males indicates that in males, the AVP participates in responses to CSDS. However, the decrease in OT/c-Fos cells in the PVN in females exposed to CSDS indicates that the OT system is more sensitive in females. These findings elucidate the mechanisms underlying the sexual differences in vulnerability and resilience to CSDS and enhance the understanding of CSDS-induced sex-biased social and emotional alterations.

## Data Availability Statement

The original contributions presented in the study are included in the article/supplementary material, further inquiries can be directed to the corresponding author/s.

## Ethics Statement

The animal study was reviewed and approved by The Animal Care and Use Committee of Shaanxi Normal University.

## Author Contributions

WH and FT designed the study. WH is the principal investigator of the study. HM, YX, and XZ performed the behavioral experiments. SH, WC, and ZH helped with Western blot and immunofluorescence experiments. ZH and RJ contributed to the statistical analysis. WH wrote the manuscript. FT and ZH revised the data and the manuscript. Part of this work was supported by a grant received by FT. All authors contributed to the article and approved the submitted version.

## Conflict of Interest

The authors declare that the research was conducted in the absence of any commercial or financial relationships that could be construed as a potential conflict of interest.

## Footnotes

1 http://www.jwatcher.ucla.edu/

## References

[B1] Anacker C. Luna V. M. Stevens G. S. Millette A. Shores R. Jimenez J. C. (2018). Hippocampal neurogenesis confers stress resilience by inhibiting the ventral dentate gyrus. *Nature* 559 98–102. 10.1038/s41586-018-0262-4 29950730 PMC6118212

[B2] Bahi A. Al Mansouri S. Al Maamari E. (2016). Nucleus accumbens lentiviral-mediated gain of function of the oxytocin receptor regulates anxiety- and ethanol-related behaviors in adult mice. *Physiol. Behav.* 164(Pt A) 249–258. 10.1016/j.physbeh.2016.06.009 27306084

[B3] Bangasser D. A. Wicks B. (2017). Sex-specific mechanisms for responding to stress. *J. Neurosci. Res.* 95 75–82. 10.1002/jnr.23812 27870416 PMC5120612

[B4] Blanchard R. J. McKittrick C. R. Blanchard D. C. (2001). Animal models of social stress: effects on behavior and brain neurochemical systems. *Physiol. Behav.* 73 261–271. 10.1016/s0031-9384(01)00449-8 11438351

[B5] Bosch O. J. Dabrowska J. Modi M. E. Johnson Z. V. Keebaugh A. C. Barrett C. E. (2016). Oxytocin in the nucleus accumbens shell reverses CRFR2-evoked passive stress-coping after partner loss in monogamous male prairie voles. *Psychoneuroendocrinology* 64 66–78. 10.1016/j.psyneuen.2015.11.011 26615473 PMC4698175

[B6] Bredewold R. Veenema A. H. (2018). Sex differences in the regulation of social and anxiety-related behaviors: insights from vasopressin and oxytocin brain systems. *Curr. Opin. Neurobiol.* 49 132–140. 10.1016/j.conb.2018.02.011 29518698 PMC6055524

[B7] Bredewold R. Smith C. J. Dumais K. M. Veenema A. H. (2014). Sex-specific modulation of juvenile social play behavior by vasopressin and oxytocin depends on social context. *Front. Behav. Neurosci.* 8:216. 10.3389/fnbeh.2014.00216 24982623 PMC4058593

[B8] Carrier N. Kabbaj M. (2012). Sex differences in social interaction behaviors in rats are mediated by extracellular signal-regulated kinase 2 expression in the medial prefrontal cortex. *Neuroscience* 212 86–92. 10.1016/j.neuroscience.2012.03.041 22521590 PMC3367089

[B9] Carter C. S. (2007). Sex differences in oxytocin and vasopressin: implications for autism spectrum disorders? *Behav. Brain Res.* 176 170–186. 10.1016/j.bbr.2006.08.025 17000015

[B10] Cavigelli S. A. Caruso M. J. (2015). Sex, social status and physiological stress in primates: the importance of social and glucocorticoid dynamics. *Philos. Trans. R Soc. Lond. B Biol. Sci.* 370:1669. 10.1098/rstb.2014.0103 25870390 PMC4410370

[B11] Dalla C. Antoniou K. Drossopoulou G. Xagoraris M. Kokras N. Sfikakis A. (2005). Chronic mild stress impact: are females more vulnerable? *Neuroscience* 135 703–714. 10.1016/j.neuroscience.2005.06.068 16125862

[B12] De Vries G. J. (2008). Sex differences in vasopressin and oxytocin innervation of the brain. *Prog. Brain Res.* 170 17–27. 10.1016/S0079-6123(08)00402-0 18655868

[B13] De Vries G. J. Panzica G. C. (2006). Sexual differentiation of central vasopressin and vasotocin systems in vertebrates: different mechanisms, similar endpoints. *Neuroscience* 138 947–955. 10.1016/j.neuroscience.2005.07.050 16310321 PMC1457099

[B14] Disner S. G. Beevers C. G. Haigh E. A. Beck A. T. (2011). Neural mechanisms of the cognitive model of depression. *Nat. Rev. Neurosci.* 12 467–477. 10.1038/nrn3027 21731066

[B15] Donovan M. Liu Y. Wang Z. (2018). Anxiety-like behavior and neuropeptide receptor expression in male and female prairie voles: the effects of stress and social buffering. *Behav. Brain Res.* 342 70–78. 10.1016/j.bbr.2018.01.015 29355675 PMC5807102

[B16] Dumais K. M. Veenema A. H. (2016). Vasopressin and oxytocin receptor systems in the brain: sex differences and sex-specific regulation of social behavior. *Front. Neuroendocrinol.* 40:3. 10.1016/j.yfrne.2015.04.003 25951955 PMC4633405

[B17] Dumais K. M. Bredewold R. Mayer T. E. Veenema A. H. (2013). Sex differences in oxytocin receptor binding in forebrain regions: correlations with social interest in brain region- and sex- specific ways. *Horm. Behav.* 64 693–701. 10.1016/j.yhbeh.2013.08.012 24055336

[B18] Duque-Wilckens N. Steinman M. Q. Busnelli M. Chini B. Yokoyama S. Pham M. (2018). Oxytocin receptors in the anteromedial bed nucleus of the stria terminalis promote stress-induced social avoidance in female California mice. *Biol. Psychiatry* 83 203–213. 10.1016/j.biopsych.2017.08.024 29066224 PMC5743604

[B19] Ehlers C. L. Kaneko W. M. Owens M. J. Nemeroff C. B. (1993). Effects of gender and social isolation on electroencephalogram and neuroendocrine parameters in rats. *Biol. Psychiatry* 33 358–366. 10.1016/0006-3223(93)90325-8 8471694

[B20] Everts H. G. Koolhaas J. M. (1999). Differential modulation of lateral septal vasopressin receptor blockade in spatial learning, social recognition, and anxiety-related behaviors in rats. *Behav. Brain Res.* 99 7–16. 10.1016/s0166-4328(98)00004-7 10512567

[B21] Fang Y. Y. Yamaguchi T. Song S. C. Tritsch N. X. Lin D. (2018). A hypothalamic midbrain pathway essential for driving maternal behaviors. *Neuron* 98 192–207.e110. 10.1016/j.neuron.2018.02.019 29621487 PMC5890946

[B22] Franklin C. L. Raines A. M. Chambliss J. L. Walton J. L. Maieritsch K. P. (2018). Examining various subthreshold definitions of PTSD using the clinician administered PTSD Scale for DSM-5. *J. Affect. Disord.* 234 256–260. 10.1016/j.jad.2018.03.001 29550742

[B23] Goodson J. L. (2005). The vertebrate social behavior network: evolutionary themes and variations. *Horm. Behav.* 48 11–22. 10.1016/j.yhbeh.2005.02.003 15885690 PMC2570781

[B24] Greenberg G. D. Steinman M. Q. Doig I. E. Hao R. Trainor B. C. (2015). Effects of social defeat on dopamine neurons in the ventral tegmental area in male and female California mice. *Eur. J. Neurosci.* 42 3081–3094. 10.1111/ejn.13099 26469289 PMC4715680

[B25] Grieb Z. A. Ross A. P. McCann K. E. Lee S. Welch M. Gomez M. G. (2020). Sex-dependent effects of social status on the regulation of arginine-vasopressin (AVP) V1a, oxytocin (OT), and serotonin (5-HT) 1A receptor binding and aggression in Syrian hamsters (Mesocricetus auratus). *Horm. Behav.* 127:104878. 10.1016/j.yhbeh.2020.104878 33148500 PMC8889570

[B26] Grippo A. J. Trahanas D. M. Zimmerman R. R. II Porges S. W. Carter C. S. (2009). Oxytocin protects against negative behavioral and autonomic consequences of long-term social isolation. *Psychoneuroendocrinology* 34 1542–1553. 10.1016/j.psyneuen.2009.05.017 19553027 PMC2841348

[B27] Haller J. Fuchs E. Halasz J. Makara G. B. (1999). Defeat is a major stressor in males while social instability is stressful mainly in females: towards the development of a social stress model in female rats. *Brain Res. Bull.* 50 33–39. 10.1016/s0361-9230(99)00087-8 10507469

[B28] He Z. Young L. Ma X. M. Guo Q. Wang L. Yang Y. (2019). Increased anxiety and decreased sociability induced by paternal deprivation involve the PVN-PrL OTergic pathway. *Elife* 8:44026. 10.7554/eLife.44026 31084703 PMC6516825

[B29] Hollis F. Kabbaj M. (2014). Social defeat as an animal model for depression. *ILAR J.* 55 221–232. 10.1093/ilar/ilu002 25225302

[B30] Holly E. N. Shimamoto A. Debold J. F. Miczek K. A. (2012). Sex differences in behavioral and neural cross-sensitization and escalated cocaine taking as a result of episodic social defeat stress in rats. *Psychopharmacology (Berl)* 224 179–188. 10.1007/s00213-012-2846-2 22926005 PMC3684960

[B31] Holt-Lunstad J. Birmingham W. Light K. C. (2011). The influence of depressive symptomatology and perceived stress on plasma and salivary oxytocin before, during and after a support enhancement intervention. *Psychoneuroendocrinology* 36 1249–1256. 10.1016/j.psyneuen.2011.03.007 21507578

[B32] Hou W. He Z. Yang Y. Yuan W. Wang L. Zhang J. (2020). The involvement of oxytocin in the effects of chronic social defeat stress on emotional behaviours in adult female mandarin voles. *Eur. J. Neurosci.* 52 2853–2872. 10.1111/ejn.14691 32011013

[B33] Huhman K. L. Solomon M. B. Janicki M. Harmon A. C. Lin S. M. Israel J. E. (2003). Conditioned defeat in male and female Syrian hamsters. *Horm. Behav.* 44 293–299. 10.1016/j.yhbeh.2003.05.001 14609551

[B34] Insel T. R. (2010). The challenge of translation in social neuroscience: a review of oxytocin, vasopressin, and affiliative behavior. *Neuron* 65 768–779. 10.1016/j.neuron.2010.03.005 20346754 PMC2847497

[B35] Insel T. R. Shapiro L. E. (1992). Oxytocin receptor distribution reflects social organization in monogamous and polygamous voles. *Proc. Natl. Acad. Sci. U.S.A.* 89 5981–5985. 10.1073/pnas.89.13.5981 1321430 PMC402122

[B36] Kessler R. C. McGonagle K. A. Swartz M. Blazer D. G. Nelson C. B. (1993). Sex and depression in the national comorbidity survey. I: lifetime prevalence, chronicity and recurrence. *J. Affect. Disord.* 29 85–96. 10.1016/0165-0327(93)90026-g 8300981

[B37] Kessler R. C. Sonnega A. Bromet E. Hughes M. Nelson C. B. (1995). Posttraumatic stress disorder in the national comorbidity survey. *Arch. Gen. Psychiatry* 52 1048–1060. 10.1001/archpsyc.1995.03950240066012 7492257

[B38] Klinsey S. G. Bailey M. T. Sheridan J. F. Padgett D. A. Avitsur R. (2007). Repeated social defeat causes increased anxiety-like behavior and alters splenocyte function in C57BL/6 and CD-1 mice. *Brain Behav. Immun.* 21 458–466. 10.1016/j.bbi.2006.11.001 17178210 PMC1941837

[B39] Koolhaas J. M. Korte S. M. De Boer S. F. Van Der Vegt B. J. Van Reenen C. G. Hopster H. (1999). Coping styles in animals: current status in behavior and stress-physiology. *Neurosci. Biobehav. Rev.* 23 925–935. 10.1016/s0149-7634(99)00026-3 10580307

[B40] Landgraf R. Neumann I. D. (2004). Vasopressin and oxytocin release within the brain: a dynamic concept of multiple and variable modes of neuropeptide communication. *Front. Neuroendocrinol.* 25:150–176. 10.1016/j.yfrne.2004.05.001 15589267

[B41] Landgraf R. Frank E. Aldag J. M. Neumann I. D. Sharer C. A. Ren X. (2003). Viral vector-mediated gene transfer of the vole V1a vasopressin receptor in the rat septum: improved social discrimination and active social behaviour. *Eur. J. Neurosci.* 18 403–411. 10.1046/j.1460-9568.2003.02750.x 12887422

[B42] Landgraf R. Gerstberger R. Montkowski A. Probst J. C. Wotjak C. T. Holsboer F. (1995). V1 vasopressin receptor antisense oligodeoxynucleotide into septum reduces vasopressin binding, social discrimination abilities, and anxiety-related behavior in rats. *J. Neurosci.* 15 4250–4258. 10.1523/jneurosci.15-06-04250.1995 7790909 PMC6577715

[B43] Lebron-Milad K. Milad M. R. (2012). Sex differences, gonadal hormones and the fear extinction network: implications for anxiety disorders. *Biol. Mood Anxiety Disord.* 2:3. 10.1186/2045-5380-2-3 22738383 PMC3384233

[B44] Li K. Nakajima M. Ibanez-Tallon I. Heintz N. (2016). A cortical circuit for sexually dimorphic oxytocin-dependent anxiety behaviors. *Cell* 167 60–72.e11. 10.1016/j.cell.2016.08.067 27641503 PMC5220951

[B45] Li L. F. Yuan W. He Z. X. Wang L. M. Jing X. Y. Zhang J. (2019). Involvement of oxytocin and GABA in consolation behavior elicited by socially defeated individuals in mandarin voles. *Psychoneuroendocrinology* 103 14–24. 10.1016/j.psyneuen.2018.12.238 30605804

[B46] Liebsch G. Wotjak C. T. Landgraf R. Engelmann M. (1996). Septal vasopressin modulates anxiety-related behaviour in rats. *Neurosci. Lett.* 217 101–104. 10.1016/0304-3940(96)13069-x 8916082

[B47] Litvin Y. Murakami G. Pfaff D. W. (2011). Effects of chronic social defeat on behavioral and neural correlates of sociality: vasopressin, oxytocin and the vasopressinergic V1b receptor. *Physiol. Behav.* 103 393–403. 10.1016/j.physbeh.2011.03.007 21397619

[B48] Lynch J. III Cullen P. K. Jasnow A. M. Riccio D. C. (2013). Sex differences in the generalization of fear as a function of retention intervals. *Learn. Mem.* 20 628–632. 10.1101/lm.032011.113 24131793

[B49] McCormick C. M. Robarts D. Kopeikina K. Kelsey J. E. (2005). Long-lasting, sex- and age-specific effects of social stressors on corticosterone responses to restraint and on locomotor responses to psychostimulants in rats. *Horm. Behav.* 48 64–74. 10.1016/j.yhbeh.2005.01.008 15919386

[B50] Nakajima M. Watanabe T. Aoki R. Shimizu R. Okuyama S. Furukawa Y. (2016). Phenotypes associated with psychiatric disorders are sex-specific in a mutant mouse line. *Brain Res.* 1652 53–61. 10.1016/j.brainres.2016.09.037 27693417

[B51] Neumann I. D. (2008). Brain oxytocin: a key regulator of emotional and social behaviours in both females and males. *J. Neuroendocrinol.* 20 858–865. 10.1111/j.1365-2826.2008.01726.x 18601710

[B52] Newman S. W. (1999). The medial extended amygdala in male reproductive behavior. A node in the mammalian social behavior network. *Ann. N. Y. Acad. Sci.* 877 242–257. 10.1111/j.1749-6632.1999.tb09271.x 10415653

[B53] Nomura M. Saito J. Ueta Y. Muglia L. J. Pfaff D. W. Ogawa S. (2003). Enhanced up-regulation of corticotropin-releasing hormone gene expression in response to restraint stress in the hypothalamic paraventricular nucleus of oxytocin gene-deficient male mice. *J. Neuroendocrinol.* 15 1054–1061. 10.1046/j.1365-2826.2003.01095.x 14622435

[B54] O’Connell L. A. Hofmann H. A. (2012). Evolution of a vertebrate social decision-making network. *Science* 336 1154–1157. 10.1126/science.1218889 22654056

[B55] Oliveira V. E. D. Neumann I. D. de Jong T. R. (2019). Post-weaning social isolation exacerbates aggression in both sexes and affects the vasopressin and oxytocin system in a sex-specific manner. *Neuropharmacology* 156:19. 10.1016/j.neuropharm.2019.01.019 30664846

[B56] Page G. G. Opp M. R. Kozachik S. L. (2016). Sex differences in sleep, anhedonia, and HPA axis activity in a rat model of chronic social defeat. *Neurobiol. Stress* 3 105–113. 10.1016/j.ynstr.2016.03.002 27981183 PMC5146204

[B57] Palanza P. Parmigiani S. (2017). How does sex matter? Behavior, stress and animal models of neurobehavioral disorders. *Neurosci. Biobehav. Rev.* 76(Pt A) 134–143. 10.1016/j.neubiorev.2017.01.037 28434584

[B58] Pierrehumbert B. Torrisi R. Laufer D. Halfon O. Ansermet F. Beck Popovic M. (2010). Oxytocin response to an experimental psychosocial challenge in adults exposed to traumatic experiences during childhood or adolescence. *Neuroscience* 166 168–177. 10.1016/j.neuroscience.2009.12.016 20018229

[B59] Raggenbass M. Tribollet E. Dubois-Dauphin M. Dreifuss J. J. (1989). Vasopressin receptors of the vasopressor (V1) type in the nucleus of the solitary tract of the rat mediate direct neuronal excitation. *J. Neurosci.* 9 3929–3936. 10.1523/jneurosci.09-11-03929.1989 2531217 PMC6569922

[B60] Rood B. D. Stott R. T. You S. Smith C. J. Woodbury M. E. De Vries G. J. (2013). Site of origin of and sex differences in the vasopressin innervation of the mouse (*Mus musculus*) brain. *J. Comp. Neurol.* 521 2321–2358. 10.1002/cne.23288 23239101

[B61] Ross A. P. McCann K. E. Larkin T. E. Song Z. Grieb Z. A. Huhman K. L. (2019). Sex-dependent effects of social isolation on the regulation of arginine-vasopressin (AVP) V1a, oxytocin (OT) and serotonin (5HT) 1a receptor binding and aggression. *Horm. Behav.* 116:104578. 10.1016/j.yhbeh.2019.104578 31449813 PMC6885541

[B62] Ross H. E. Cole C. D. Smith Y. Neumann I. D. Landgraf R. Murphy A. Z. (2009). Characterization of the oxytocin system regulating affiliative behavior in female prairie voles. *Neuroscience* 162 892–903. 10.1016/j.neuroscience.2009.05.055 19482070 PMC2744157

[B63] Silva A. L. Fry W. H. Sweeney C. Trainor B. C. (2010). Effects of photoperiod and experience on aggressive behavior in female California mice. *Behav. Brain Res.* 208 528–534. 10.1016/j.bbr.2009.12.038 20060017 PMC2831116

[B64] Smeltzer M. D. Curtis J. T. Aragona B. J. Wang Z. (2006). Dopamine, oxytocin, and vasopressin receptor binding in the medial prefrontal cortex of monogamous and promiscuous voles. *Neurosci. Lett.* 394 146–151. 10.1016/j.neulet.2005.10.019 16289323

[B65] Solomon M. B. Karom M. C. Huhman K. L. (2007). Sex and estrous cycle differences in the display of conditioned defeat in Syrian hamsters. *Horm. Behav.* 52 211–219. 10.1016/j.yhbeh.2007.04.007 17555756

[B66] Steinman M. Q. Trainor B. C. (2017). Sex differences in the effects of social defeat on brain and behavior in the California mouse: insights from a monogamous rodent. *Semin. Cell Dev. Biol.* 61 92–98. 10.1016/j.semcdb.2016.06.021 27375045 PMC5201444

[B67] Steinman M. Q. Duque-Wilckens N. Greenberg G. D. Hao R. Campi K. L. Laredo S. A. (2016). Sex-specific effects of stress on oxytocin neurons correspond with responses to intranasal oxytocin. *Biol. Psychiatry* 80 406–414. 10.1016/j.biopsych.2015.10.007 26620251 PMC4837091

[B68] Steinman M. Q. Laredo S. A. Lopez E. M. Manning C. E. Hao R. C. Doig I. E. (2015). Hypothalamic vasopressin systems are more sensitive to the long term effects of social defeat in males versus females. *Psychoneuroendocrinology* 51 122–134. 10.1016/j.psyneuen.2014.09.009 25306217 PMC4268083

[B69] Stoop R. (2012). Neuromodulation by oxytocin and vasopressin. *Neuron* 76 142–159. 10.1016/j.neuron.2012.09.025 23040812

[B70] Takayanagi Y. Yoshida M. Bielsky I. F. Ross H. E. Kawamata M. Onaka T. (2005). Pervasive social deficits, but normal parturition, in oxytocin receptor-deficient mice. *Proc. Natl. Acad. Sci. U.S.A.* 102 16096–16101. 10.1073/pnas.0505312102 16249339 PMC1276060

[B71] Trainor B. C. Pride M. C. Villalon Landeros R. Knoblauch N. W. Takahashi E. Y. Silva A. L. (2011). Sex differences in social interaction behavior following social defeat stress in the monogamous California mouse (*Peromyscus californicus*). *PLoS One* 6:e17405. 10.1371/journal.pone.0017405 21364768 PMC3045459

[B72] Veenema A. H. Beiderbeck D. I. Lukas M. Neumann I. D. (2010). Distinct correlations of vasopressin release within the lateral septum and the bed nucleus of the stria terminalis with the display of intermale aggression. *Horm. Behav.* 58 273–281. 10.1016/j.yhbeh.2010.03.006 20298693

[B73] Veenema A. H. Bredewold R. Neumann I. D. (2007). Opposite effects of maternal separation on intermale and maternal aggression in C57BL/6 mice: link to hypothalamic vasopressin and oxytocin immunoreactivity. *Psychoneuroendocrinology* 32 437–450. 10.1016/j.psyneuen.2007.02.008 17433558

[B74] Venzala E. Garcia-Garcia A. L. Elizalde N. Delagrange P. Tordera R. M. (2012). Chronic social defeat stress model: behavioral features, antidepressant action, and interaction with biological risk factors. *Psychopharmacology (Berl)* 224 313–325. 10.1007/s00213-012-2754-5 22707231

[B75] Walsh J. J. Christoffel D. J. Heifets B. D. Ben-Dor G. A. Selimbeyoglu A. Hung L. W. (2018). 5-HT release in nucleus accumbens rescues social deficits in mouse autism model. *Nature* 560 589–594. 10.1038/s41586-018-0416-4 30089910 PMC8164568

[B76] Wang L. Hou W. He Z. Yuan W. Yang J. Yang Y. (2018). Effects of chronic social defeat on social behaviors in adult female mandarin voles (*Microtus mandarinus*): involvement of the oxytocin system in the nucleus accumbens. *Prog. Neuropsychopharmacol. Biol. Psychiatry* 82 278–288. 10.1016/j.pnpbp.2017.11.002 29126982

[B77] Weller J. E. Camerlink I. Turner S. P. Farish M. Arnott G. (2019). Socialisation and its effect on play behaviour and aggression in the domestic pig (*Sus scrofa*). *Sci. Rep.* 9:4180. 10.1038/s41598-019-40980-1 30862880 PMC6414639

[B78] Young L. J. (1999). Frank A. Beach award. Oxytocin and vasopressin receptors and species-typical social behaviors. *Horm. Behav.* 36 212–221. 10.1006/hbeh.1999.1548 10603285

[B79] Yuan W. He Z. Hou W. Wang L. Li L. Zhang J. (2019). Role of oxytocin in the medial preoptic area (MPOA) in the modulation of paternal behavior in mandarin voles. *Horm. Behav.* 110 46–55. 10.1016/j.yhbeh.2019.02.014 30836063

[B80] Yuan W. Li L. Hou W. He Z. Wang L. Zhang J. (2020). Preweaning paternal deprivation impacts parental responses to pups and alters the serum oxytocin and corticosterone levels and oxytocin receptor, vasopressin 1A receptor, oestrogen receptor, dopamine type I receptor, dopamine type II receptor levels in relevant brain regions in adult mandarin voles. *Neuroendocrinology* 110 292–306. 10.1159/000501798 31256151

